# Mode and Polarization Division Multiplexing Based on Chalcogenide‐Glass‐on‐Thin‐Film Lithium Niobate

**DOI:** 10.1002/nap2.70115

**Published:** 2026-05-05

**Authors:** Zhexi Chen, Xin Hu, Yelu Shen, Lei Yang, Zhiqiang Shen, Yingxuan Zhao, Peipeng Xu, Zhen Sheng, Fuwan Gan

**Affiliations:** ^1^ Faculty of Electrical Engineering and Computer Science Ningbo University Ningbo China; ^2^ State Key Laboratory of Functional Materials for Informatics Shanghai Institute of Microsystem and Information Technology Chinese Academy of Sciences Shanghai China

## Abstract

We report integrated mode‐ and polarization‐division multiplexing components on a high‐index‐contrast Ge_28_Sb_12_Se_60_ (GeSbSe) chalcogenide‐glass‐loaded thin‐film lithium niobate on insulator (LNOI) platform operating across the 1500–1570 nm wavelength range. By leveraging the direction‐dependent birefringence of x‐cut LNOI together with the strong optical confinement provided by the GeSbSe overlayer, we experimentally demonstrate a four‐channel mode (de)multiplexer (MDM) and an asymmetric‐directional‐coupler‐based polarization splitter–rotator (PSR). Over the full band, the MDM achieves insertion losses below 0.97, 0.31, 2.36, and 3.21 dB for the TE_0_–TE_3_ channels, with intermodal crosstalk better than −18.3, −19.2, −14.9, and −16.3 dB, respectively. The PSR exhibits insertion losses of 1.67 dB (TM_0_ input) and 1.81 dB (TE_0_ input), together with extinction ratios exceeding 20.7 and 31.6 dB. These results validate GeSbSe‐on‐LNOI as a scalable platform for broadband low‐loss multidimensional multiplexing in high‐capacity photonic integrated circuits.

## Graphical abstract

High‐index GeSbSe chalcogenide glass loaded on thin‐film lithium niobate enables compact broadband mode‐ and polarization‐division multiplexing. Exploiting direction‐dependent birefringence of x‐cut LNOI, a four‐channel mode (de)multiplexer and asymmetric directional coupler‐based polarization splitter–rotator are demonstrated with great performance in the 1500–1570 nm band. This platform advances multidimensional photonic integration.

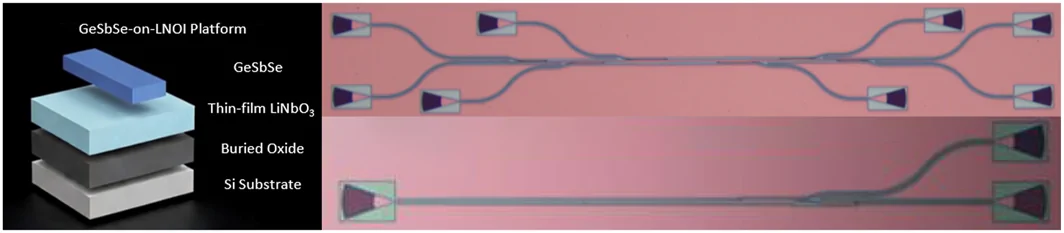

## 1 Introduction

Global data traffic continues to grow rapidly, driven by cloud computing, artificial intelligence (AI), and bandwidth‐hungry optical interconnects. This trend is accelerating the demand for photonic integrated circuits (PICs) that offer higher bandwidth density, lower power consumption, and increased functional integration [1, 2, 3]. Spatial multiplexing, particularly mode‐division multiplexing (MDM) and polarization‐division multiplexing (PDM), provides an efficient route to scaling link capacity without consuming additional wavelength resources, thereby reducing spectral congestion and system cost [4, 5, 6].

MDM and PDM components have been extensively developed on silicon‐on‐insulator (SOI) due to its strong index contrast and mature fabrication ecosystem [7, 8]. However, high‐speed electro‐optic (EO) modulation in silicon typically relies on the plasma‐dispersion effect, which imposes intrinsic trade‐offs among modulation efficiency, bandwidth, loss, and nonlinearity‐induced penalties [9, 10]. Thin‐film lithium niobate on insulator (LNOI) has therefore emerged as a leading PIC platform, combining a broad transparency window with a large Pockels coefficient (*r*_33_) and enabling ultra‐high‐speed, low‐voltage modulators [11, 12, 13, 14]. In parallel, recent advances in rare‐earth‐ion‐doped thin‐film lithium niobate have further expanded the functionality of the LNOI platform toward active photonic devices. Integrated lasers and waveguide amplifiers based on Er^3+^‐ and Yb^3+^‐doped thin‐film lithium niobate have demonstrated strong potential for on‐chip light sources, optical amplification, and multifunctional photonic integration [15, 16, 17, 18, 19, 20]. Despite these advantages, directly etched LNOI waveguides still face process constraints—such as limited etch depth, sidewall angle, and etch‐induced roughness—that restrict minimum gaps and complicate strongly coupled structures (e.g., directional couplers and multimode converters) [6, 21].

Dielectrically loaded LNOI platforms (e.g., Si_3_N_4_‐ or silicon‐rich‐nitride‐loaded LN) can mitigate the need for aggressive LN etching and improve fabrication tolerance, but their moderate refractive indices (*n* ≈ 2.0) lead to larger bending radii and reduced device compactness [22, 23, 24, 25]. Representative examples of multiplexing on such material‐loaded TFLN platforms include mode‐ and polarization‐division‐multiplexing devices based on asymmetric directional couplers and hybrid silicon/lithium‐niobate thin‐film waveguides [5, 25]. These studies highlight the utility of material loading for realizing multiplexing functionalities on TFLN while also indicating the footprint limitations associated with relatively low index contrast. In contrast, chalcogenide glass, particularly Ge_28_Sb_12_Se_60_ (GeSbSe), provides a high‐index alternative for LNOI loading, offering *n* ≈ 2.8 at 1550 nm together with wide transparency and strong optical confinement [26, 27, 28]. This stronger confinement supports a more compact route toward multidimensional multiplexing devices while maintaining competitive loss and crosstalk/extinction‐ratio performance.

Here we exploit the anisotropy‐engineered modal landscape of the GeSbSe‐on‐LNOI platform to realize compact mode and polarization multiplexing over the 1500–1570 nm band. We first design a four‐channel MDM based on z‐propagating waveguides, where TE and TM mode families remain well separated and free of TE–TM hybridization, enabling clean TE‐mode phase matching and low intermodal interference. We then implement a PSR on y‐propagating waveguides by intentionally harnessing TE–TM hybridization, combining adiabatic mode evolution in a tapered section with asymmetric directional coupling for robust polarization discrimination. Both devices are fabricated and experimentally characterized across the full band, demonstrating broadband operation with low loss and low crosstalk.

## 2 Principle and Design

Figure 1 compares the modal characteristics of GeSbSe‐loaded LNOI waveguides for propagation along the crystallographic *y* and *z* directions of an x‐cut LiNbO_3_ thin film. The device geometry follows our previously reported GeSbSe‐on‐LNOI platform [29], where a 400‐nm‐thick x‐cut LiNbO_3_ layer is bonded on a 2‐μm buried oxide and overlaid with a 200‐nm‐thick GeSbSe strip that provides strong index guiding. Because LiNbO_3_ is a uniaxial crystal, light polarized along the crystallographic z‐axis experiences the extraordinary refractive index (*n*_e_ ≈ 2.138), whereas polarization in the x–y plane experiences the ordinary index (*n*_0_≈2.211) at 1550 nm. This intrinsic birefringence, combined with the vertical asymmetry of the GeSbSe‐loaded geometry, produces a direction‐dependent dispersion landscape that can be exploited for either hybridization‐free multimode routing or engineered polarization manipulation.

**FIGURE 1:**
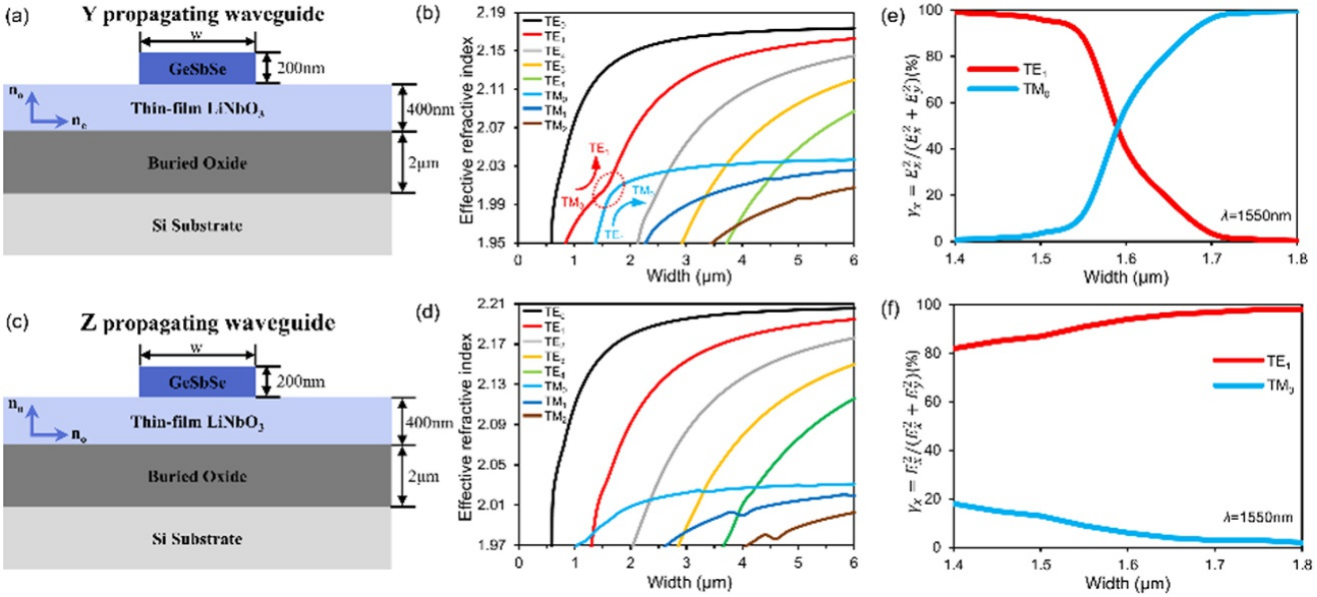
Cross‐sectional views of the GeSbSe‐on‐LNOI waveguides for (a) y propagation and (d) z propagation. Calculated effective refractive indices of the guided TE‐ and TM‐like modes versus waveguide width for the (b) y‐propagating and (e) z‐propagating waveguides. Calculated polarization fractions at 1550 nm for the relevant modes in the (c) y‐propagating and (f) z‐propagating waveguides, showing strong TE–TM hybridization in the y‐propagating case and suppressed hybridization in the z‐propagating case.

For y‐propagating waveguides as shown in Figure 1a, TE‐like modes predominantly sample *n*_e_, whereas TM‐like modes predominantly sample *n*_0_, resulting in lower effective indices for TE modes than for TM modes. The interplay between anisotropy and hybrid waveguide asymmetry creates pronounced TE–TM hybridization (anti‐crossing) regions, visible in Figure 1b around widths of ∼1.5–2.0 μm (e.g., the TE_1_–TM_0_ interaction). This behavior is further confirmed by the polarization–fraction analysis in Figure 1c, which shows a clear exchange of modal polarization character across the anticrossing region. Such hybridization can induce unintended mode mixing in multimode circuits when operating along the y direction, but it can also be harnessed for polarization conversion and splitting. At the same time, it is highly advantageous for polarization‐handling functions, as it enables controlled TE↔TM conversion, polarization rotation, and broadband polarization splitting that are difficult to realize in isotropic platforms.

In contrast, z‐propagating waveguides exhibit a markedly different modal behavior as shown in Figure 1d. For the unloaded x‐cut LNOI case, the transverse xy‐plane is effectively isotropic; therefore, the TE‐like and TM‐like modes should not be simply interpreted in terms of different ordinary and extraordinary refractive indices. Instead, after introducing the high‐index GeSbSe overlayer, the effective index landscape is strongly reshaped by the combined effects of high‐index loading and waveguide anisotropy. As shown in Figure 1e, the TE‐ and TM‐like mode families become well separated over the width range considered, and no evident TE–TM hybridization is observed. This is further confirmed by the polarization‐fraction analysis in Figure 1f, where the modal character remains strongly dominated by a single polarization without the exchange behavior seen in the y‐propagating case. This clean modal separation suppresses polarization‐induced mixing and provides a robust basis for low‐crosstalk multimode operation.

These results lead to a practical anisotropy‐driven design guideline for the GeSbSe‐on‐LNOI platform. Z‐propagation provides a robust, hybridization‐free modal environment that is well suited to low‐loss, low‐crosstalk MDM components. In contrast, y‐propagation naturally offers accessible TE–TM hybridization, which can be harnessed to realize compact polarization splitters, rotators, and hybrid‐mode‐engineered elements. This directional distinction forms the physical basis for the mode‐ and polarization‐management devices presented below.

### 2.1 Mode (De)Multiplexer

Figure 2a shows the layout of the GeSbSe‐loaded LNOI mode (de)multiplexer, oriented along the crystallographic z direction to ensure hybridization‐free TE‐mode operation. As illustrated in Figure 2b, the device employs a sequence of waveguide widths (*w*_0_ = 1 μm, *w*_1_ = 2.2 μm, *w*_2_ = 3.4 μm, and *w*_3_ = 4.6 μm) selected from simulated phase‐matching conditions for the TE_0_–TE_1_, TE_0_–TE_2_, and TE_0_–TE_3_ mode pairs. Smooth width transitions and a small inter‐arm gap (*w*_gap_ = 0.2 μm) are used to tailor the coupling strength and maintain high‐purity modal evolution. The optimized coupling lengths are *L*_1_ = 40 μm for the TE_0_–TE_1_ converter, *L*_2_ = 54 μm for the TE_0_–TE_2_ converter, and *L*_3_ = 62 μm for the TE_0_–TE_3_ converter; these sections are applied symmetrically for multiplexing and demultiplexing to ensure consistent modal selectivity across the full structure.

**FIGURE 2:**
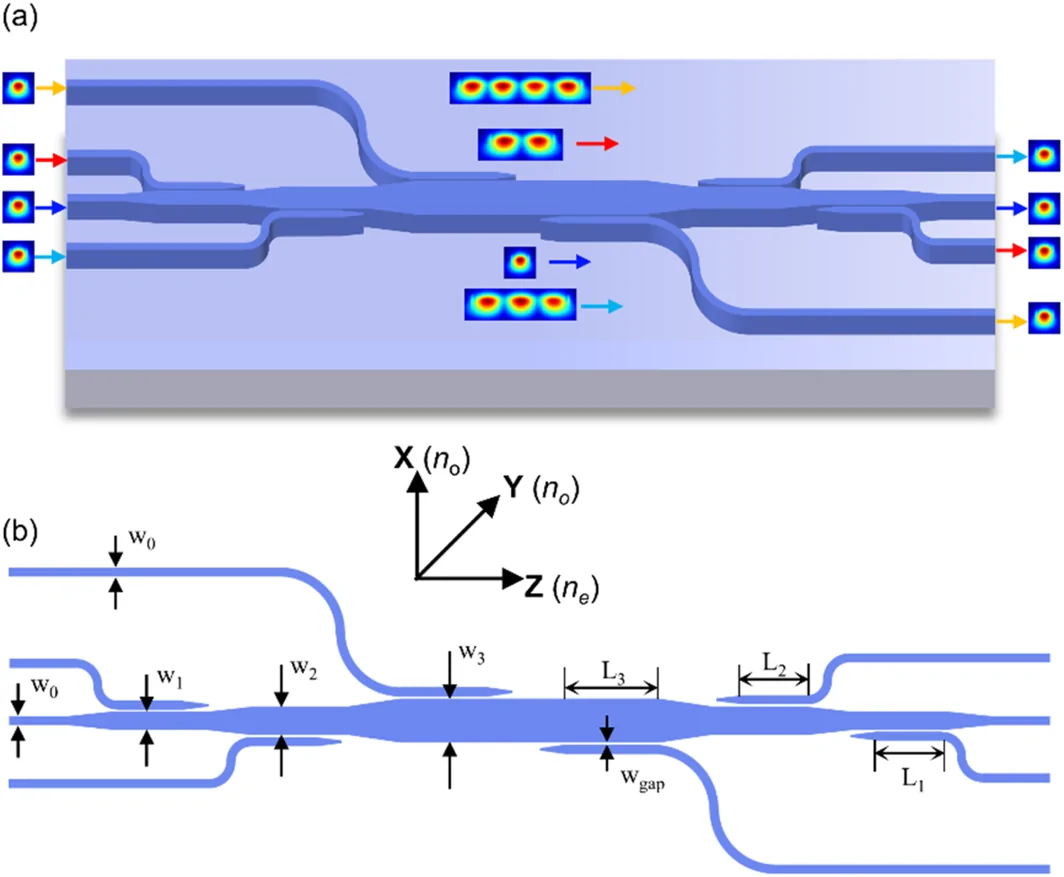
(a) The proposed mode multiplexing device consists of a multichannel mode (de)multiplexer based on z‐propagation waveguides. (b) Top view of the proposed four‐channel mode (de)multiplexer.

Figure 3 summarizes the simulated performance of the individual mode converters. For the TE_0_→TE_1_ converter, as shown in Figure 3a, the target channel exhibits < 0.54 dB insertion loss over 1500–1570 nm, whereas undesired coupling to TE_2_ is suppressed below −35 dB. The field evolution in Figure 3b confirms a clean transition from the single‐lobed TE_0_ profile to the antisymmetric TE_1_ mode. For the TE_0_→TE_2_ converter, as illustrated in Figure 3c, the insertion loss remains < 0.45 dB and the crosstalk into TE_1_ and TE_3_ stays below −26.9 dB across the band, with Figure 3d showing recovery of the two‐lobed TE_2_ pattern. For the TE_0_→TE_3_ converter as shown in Figure 3e the insertion loss is < 0.43 dB and coupling to TE_1_/TE_2_ is below −22.4 dB, whereas Figure 3f verifies high‐purity formation of the three‐lobed TE_3_ mode. These results indicate that the optimized coupling lengths support robust higher‐order mode conversion over a broad bandwidth.

**FIGURE 3:**
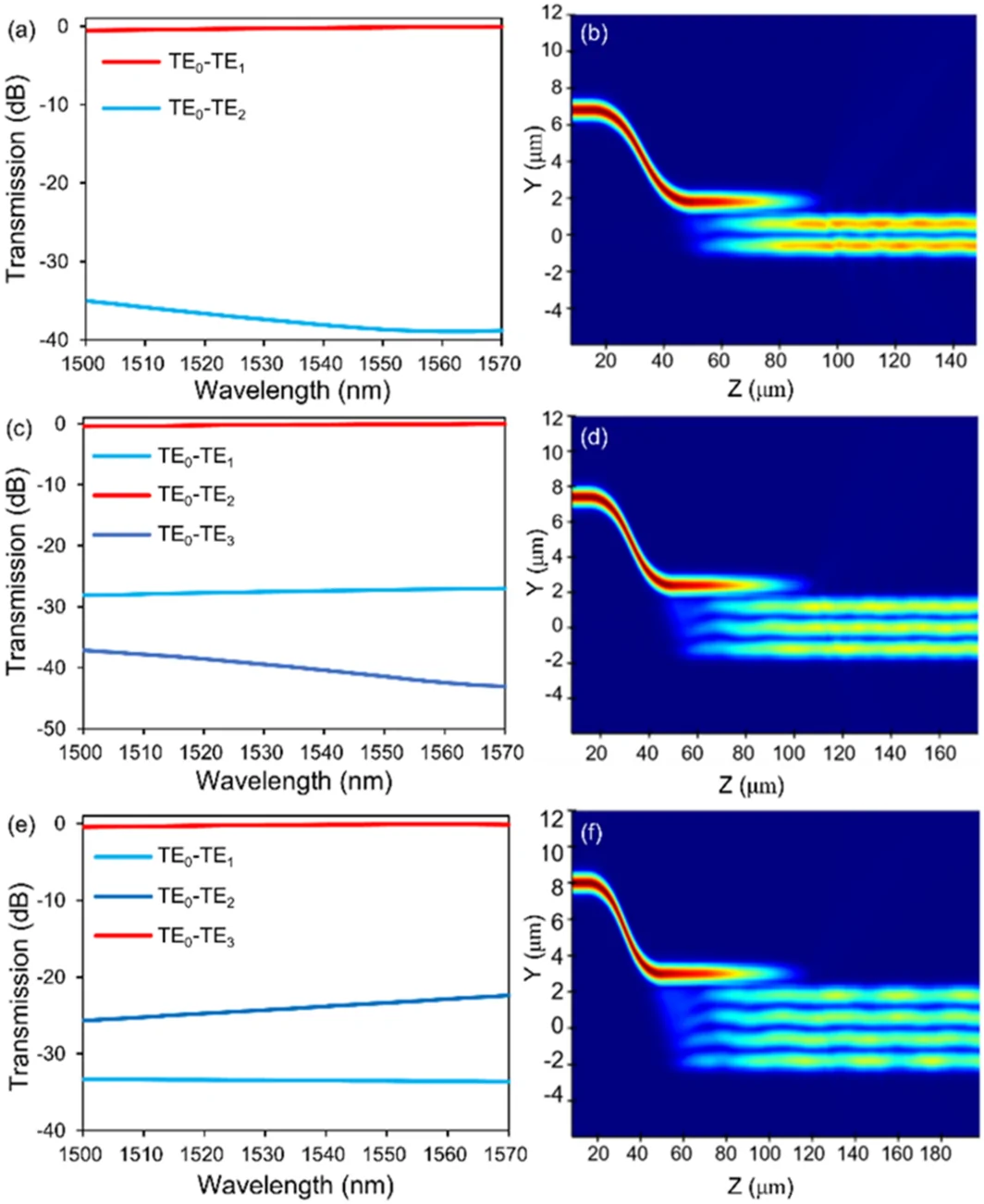
(a, c, e) Simulated normalized transmission spectra for the TE_0_‐to‐TE_1_, TE_0_‐to‐TE_2_, and TE_0_‐to‐TE_3_ conversions. (b, d, f) Corresponding simulated light propagation and E‐field profiles illustrating the evolution of the TE_0_ input into the TE_1_, TE_2_, and TE_3_ modes.

Figure 4a shows an optical microscope image of the fabricated MDM device. The device was fabricated on the GeSbSe‐on‐LNOI platform using the process reported in our previous work [29], including thermal evaporation of a 200‐nm‐thick GeSbSe film, electron‐beam lithography, and CHF_3_/CF_4_‐based ICP etching. The propagation loss of the GeSbSe‐loaded waveguides on this platform was previously characterized to be ∼3 dB/cm at 1550 nm. This dielectrically loaded architecture avoids deep etching of the LN layer and relaxes fabrication constraints. Grating couplers were co‐fabricated to enable fiber‐to‐chip coupling during characterization. The final device integrates four input ports (I_0_–I_3_) and four output ports (O_0_–O_3_) to route and interrogate four spatial modes.

**FIGURE 4:**
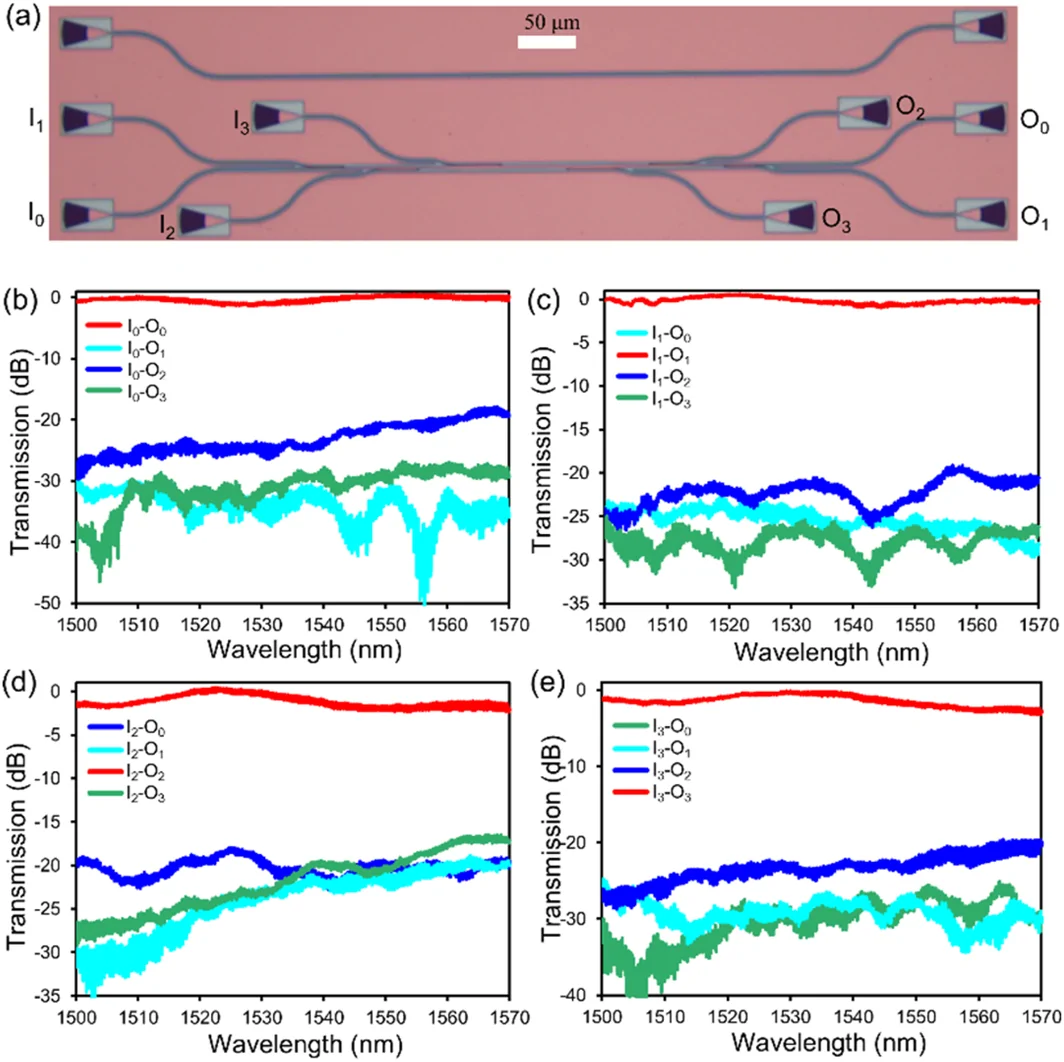
(a) Optical microscope image of the fabricated four‐channel MDM. Measured normalized transmission spectra at output ports O_0_–O_3_ for excitation at input ports (b) I_0_, (c) I_1_, (d) I_2_, and (e) I_3_. The measured spectra are normalized to the transmission of on‐chip reference straight waveguides with identical grating couplers.

Figure 4b,e plot the measured transmission spectra from 1500 to 1570 nm for excitation at ports I_0_, I_1_, I_2_, and I_3_, respectively. From these measurements, the insertion losses for the TE_0_, TE_1_, TE_2_, and TE_3_ channels are determined to be < 0.97 dB, < 0.31 dB, < 2.36 dB, and < 3.21 dB, whereas the corresponding intermodal crosstalk levels are below −18.3 dB, −19.2 dB, −14.9 dB, and −16.3 dB, respectively. The higher insertion losses of the TE_2_ and TE_3_ channels mainly result from incomplete mode evolution in the intentionally shortened higher‐order coupling regions adopted for compactness. Simulations suggest that extending these sections to > 100 μm would reduce the excess loss while preserving intermodal crosstalk below ∼−15 dB, albeit with a substantially increased footprint. Thus, the present design reflects a practical trade‐off between compactness and conversion performance.

### 2.2 Polarization (De)Multiplexer

Figure 5a illustrates the proposed polarization splitter–rotator (PSR), comprising a tapered multimode section followed by two asymmetric directional‐coupler (ADC) regions. The top‐view layout with structural parameters is shown in Figure 5b. The input single‐mode waveguide (*w*_4_ = 1 μm) is linearly tapered over *L*_4_ = 20 μm to a multimode width *w*_5_ = 1.35 μm, and then propagates through a straight multimode section of length *L*_5_ = 250 μm to enable mode evolution. The main waveguide is further expanded to *w*_6_ = 2 μm over *L*_6_ = 42 μm, forming the first ADC, where asymmetric coupling to the upper adjacent waveguide enables polarization‐rotated transfer to the cross port. Downstream, a second ADC is formed between the main waveguide and a lower adjacent waveguide over an interaction length *L*_7_ = 30 μm with a coupling gap *w*_gap_ = 0.2 μm. In this section, the main waveguide passes through an intermediate widened segment (*w*_8_ = 1.3 μm) before tapering to the final single‐mode width *w*_9_ = 1 μm at the through port, where this ADC functions as a mode filter to suppress residual TM_0_ components.

**FIGURE 5:**
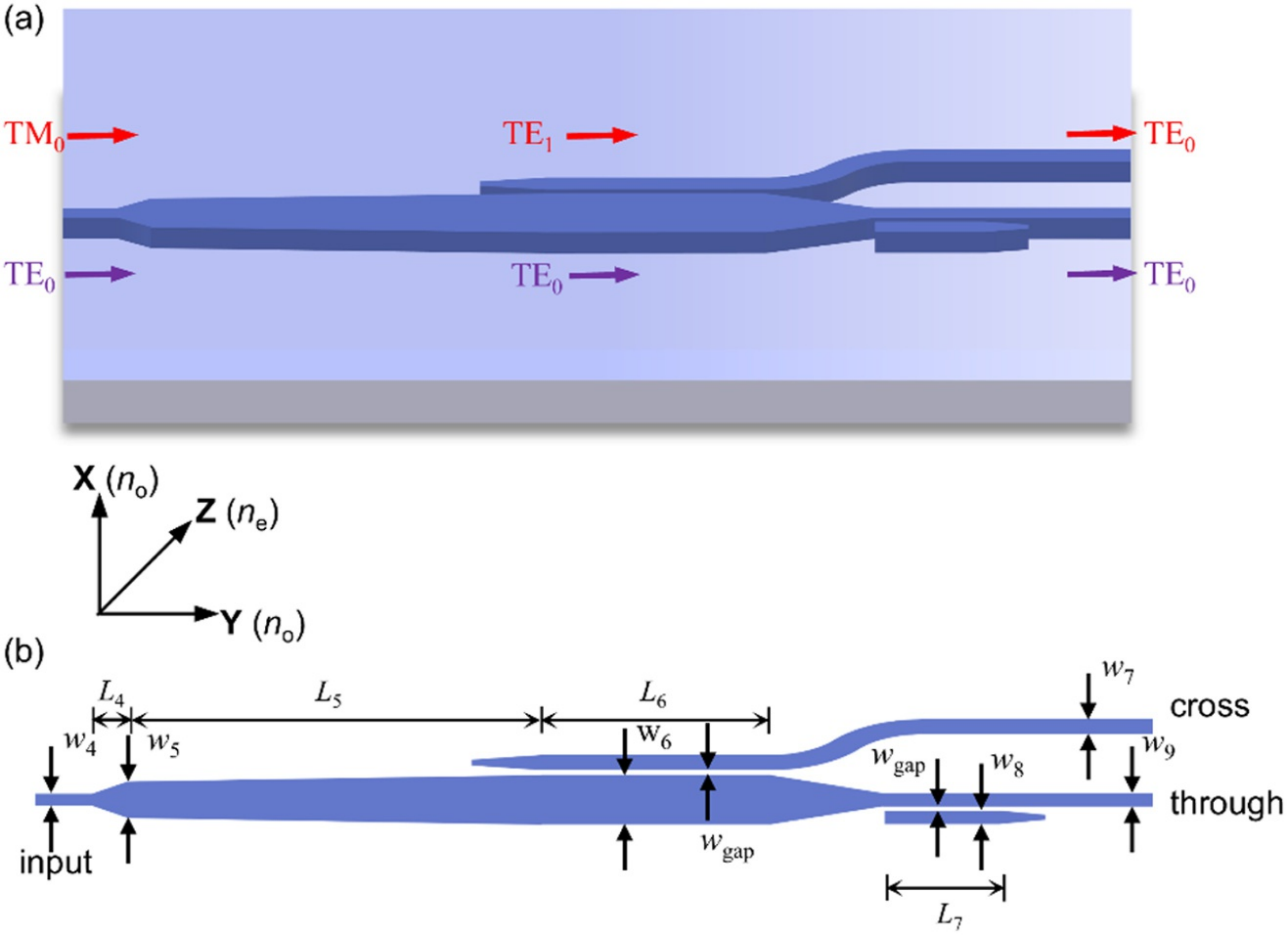
(a) 3D illustration of the ADC‐based polarization splitter–rotator. (b) Top‐view representation of the device with annotated structural dimensions.

The operating principle is as follows. For TE_0_ input, the optical field remains confined in the GeSbSe–LN hybrid core and propagates along the through path with negligible coupling due to phase mismatch with the adjacent waveguide. In contrast, for TM_0_ input, the tapered multimode section drives the mode evolution toward a TE_1_‐like profile. In the ADC region, phase matching between this TE_1_‐like mode in the main waveguide and the TE_0_ mode of the adjacent waveguide enables efficient power transfer to the cross port, yielding a polarization‐rotated TE_0_ output. In the optimized design, the adiabatic taper is designed to provide highly efficient TM_0_‐to‐TE_1_‐like conversion before the asymmetric directional coupler. This mode evolution supports a high polarization purity at the cross port and is consistent with the measured high extinction ratio. Any residual TM_0_ component that continues along the through path is subsequently suppressed by the ADC formed over *L*_7_, with the widened section *w*_8_ acting as a mode‐filtering segment, thereby strongly reducing TM_0_ leakage and ensuring high polarization discrimination.

Figure 6a,b show the simulated normalized transmission spectra of the PSR for TM_0_ and TE_0_ inputs, respectively, over 1500–1570 nm. Figure 6c,d plot the corresponding electric‐field distributions at 1550 nm. For TM_0_ excitation, the simulated insertion loss is < 0.32 dB and the extinction ratio between the cross and through ports exceeds 19.9 dB. For TE_0_ excitation, the insertion loss remains < 0.31 dB with an extinction ratio above 28.1 dB. These results confirm that the proposed structure enables broadband TE_0_/TM_0_ discrimination with low loss and high modal selectivity in simulation.

**FIGURE 6:**
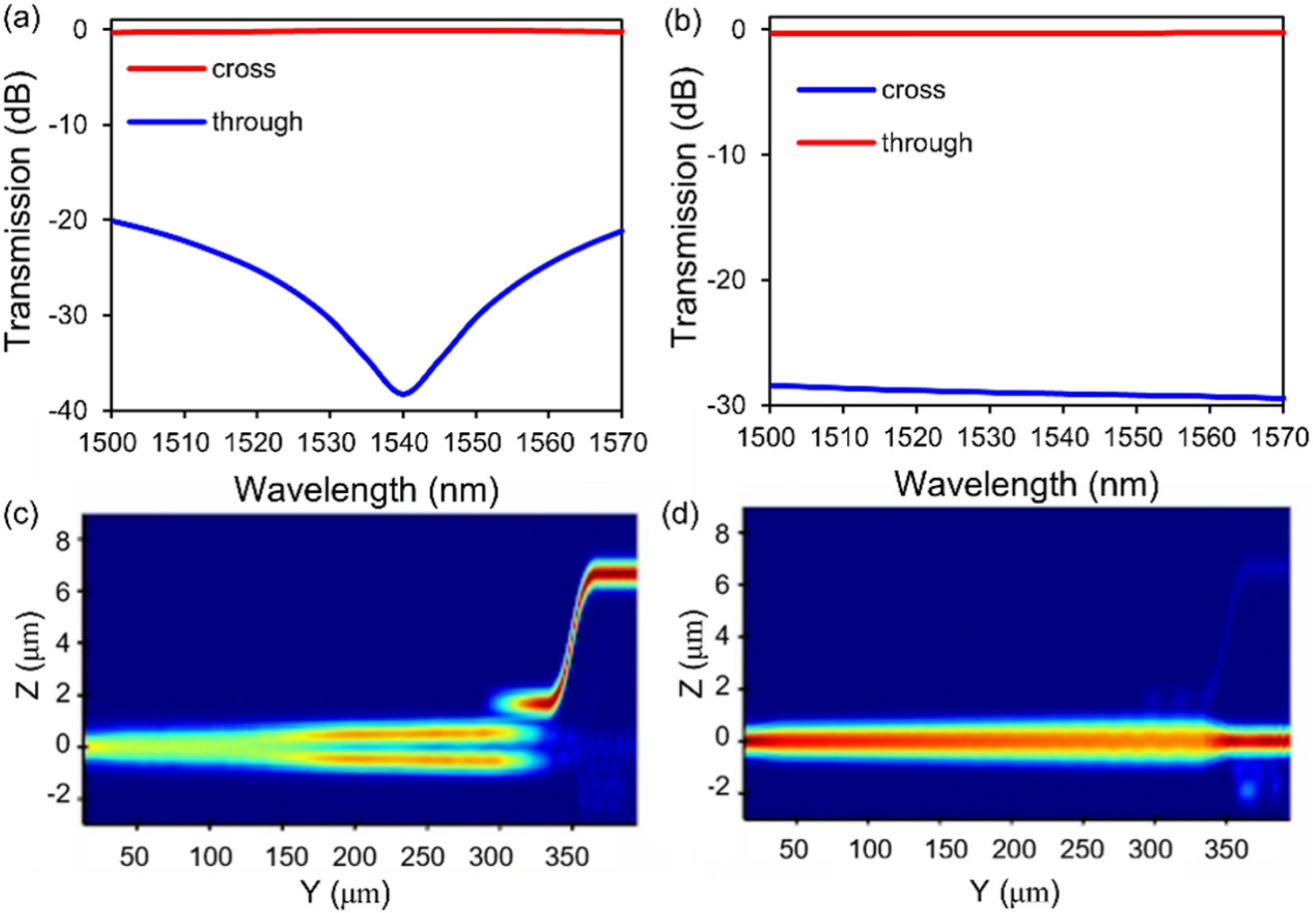
Simulated normalized transmission spectra of the PSR for (a) TM_0_ input and (b) TE_0_ input, together with the corresponding electric‐field distributions at 1550 nm for (c) TM_0_ excitation and (d) TE_0_ excitation.

Figure 7b,c present the measured normalized transmission spectra for TM_0_ and TE_0_ excitation over 1500–1570 nm, where normalization is performed using on‐chip TE/TM grating couplers. Over the band, the measured insertion loss is 1.67 dB for TM_0_ input, with an extinction ratio exceeding 20.7 dB, and 1.81 dB for TE_0_ input, with an extinction ratio above 31.6 dB. These insertion losses are higher than the simulated values (< 0.32 dB), mainly due to the cumulative propagation loss of the GeSbSe‐loaded waveguides on the GeSbSe‐on‐LNOI platform, which is approximately 3 dB/cm at 1550 nm [29]. Additional excess loss may arise from non‐ideal adiabatic mode evolution in the tapered section, fabrication‐induced dimensional deviations in the ADC regions that perturb phase matching, and scattering or residual mode mismatch in the filtering section. Nevertheless, the ADC‐assisted filtering significantly improves polarization discrimination, particularly for TM_0_ input. Further optimization of the taper, ADC, and filtering sections is expected to reduce excess loss while maintaining high extinction performance.

**FIGURE 7:**
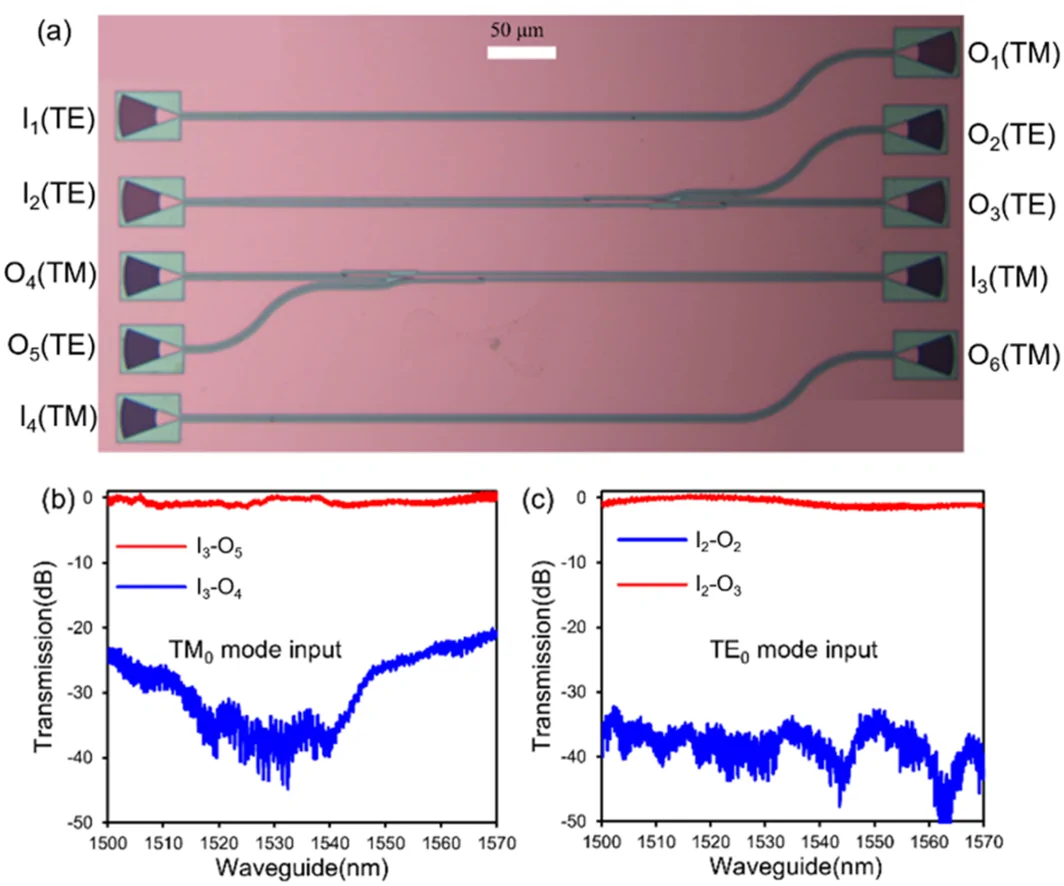
(a) Optical microscope image of the fabricated PSR. Measured normalized transmission spectra at the through and cross ports for (b) TM_0_ input and (c) TE_0_ input. The measured spectra are normalized to the peak transmission of the corresponding on‐chip TE/TM grating‐coupler reference waveguides.

## 3 Discussion and Conclusion

As summarized in Table 1, the demonstrated GeSbSe‐on‐LNOI devices achieve performance comparable to that of recent material‐loaded or hybrid‐LNOI‐multiplexing devices in terms of insertion loss, crosstalk/extinction ratio, and operating bandwidth. More importantly, the high refractive index of GeSbSe provides stronger optical confinement than moderate‐index loading materials, which is advantageous for footprint reduction and compact multidimensional integration. Combined with the propagation‐direction‐dependent birefringence of x‐cut LNOI, this platform enables both low‐crosstalk mode multiplexing and efficient polarization handling on the same chip.

**TABLE 1:** Comparison of representative recent MDM and PDM devices on LNOI‐based and hybrid platforms.

Ref.	Platform	IL (dB)	CT (dB)	ER (dB)	Bandwidth (nm)	Type
[5]	Si_3_N_4_‐loaded LNOI	1.49	−13	∼	40	MDM
[6]	Thin‐film LNOI	0.2	−19	∼	80	MDM
This work	GeSbSe‐on‐LNOI	3.21	−14.9	∼	70	MDM
[5]	Si_3_N_4_‐loaded LNOI	1.49	∼	17.75	40	PSR
[30]	Thin‐film LNOI	< 1	∼	19.6	60	PSR
This work	GeSbSe‐on‐LNOI	1.81	∼	20.7	70	PSR

*Note:* Data are taken at or near 1550 nm unless otherwise noted.

We demonstrated broadband mode‐ and polarization‐division multiplexing components on a GeSbSe‐on‐LNOI hybrid platform with stable operation across 1500–1570 nm. The four‐channel MDM achieves insertion losses of 0.97–3.21 dB for TE_0_–TE_3_ channels, with intermodal crosstalk below −14.9 dB, whereas the PSR exhibits insertion losses of 1.67 dB for TM_0_ input and 1.81 dB for TE_0_ input, together with extinction ratios exceeding 20.7 and 31.6 dB, respectively. By combining z‐propagation for hybridization‐free multimode routing and y‐propagation for engineered polarization conversion, this work highlights the synergy between LN birefringence and chalcogenide‐based index engineering for compact multidimensional multiplexing on a single LNOI chip.

## Author Contributions

**Zhexi Chen:** conceptualization, investigation, methodology, validation, software, formal analysis, supervision, data curation, visualization, writing – review and editing, writing – original draft. **Xin Hu:** methodology, validation, formal analysis. **Yelu Shen:** conceptualization, methodology, software. **Lei Yang:** methodology, validation. **Zhiqiang Shen:** methodology, software. **Yingxuan Zhao:** supervision, writing – review and editing. **Peipeng Xu:** methodology, conceptualization, funding acquisition, writing – original draft, writing – review and editing, project administration, resources, data curation, supervision. **Zhen Sheng:** supervision, writing – review and editing. **Fuwan Gan:** writing – review and editing, supervision.

## Funding

Natural Science Foundation of Zhejiang Province (Grant LZ24F050001); Shanghai Natural Science Foundation (Grant 25ZR1401379); National Natural Science Foundation of China (Grant 62305367); National Major Scientific Research Instrument Development Project (Grant 22127901).

## Data Availability

The data that support the findings of this study are available from the corresponding author upon reasonable request.
